# Understanding the role, barriers, and motivators of takeaway food purchasing and consumption in English families across socioeconomic positions: a secondary qualitative analysis

**DOI:** 10.1186/s12889-026-26585-0

**Published:** 2026-02-12

**Authors:** Aleks Saunders, Charlotte Gallagher-Squires, Paul Coleman, Rana Conway

**Affiliations:** 1 Research Department of Behavioural Science and Health, University College London, Gower Street, London, WC1E 6BT UK; 2 Centre for Food Policy, University of London, City St George’s, London, UK

**Keywords:** Health equity, Food environment, Food insecurity, Public health policy, Social determinants of health, Commercial determinants of health, Dietary consumption, Fast foods

## Abstract

**Background:**

Rates of obesity are rising globally, with socioeconomic inequalities driving this disproportionately in lower income communities. The COVID-19 pandemic and ongoing cost-of-living crisis have further exacerbated unhealthy eating patterns, including a rise in the ‘out of home’ sector. Understanding these food dynamics in families is crucial for informing equitable obesity prevention policies, particularly given than takeaway foods are often calorie dense, lacking key nutrients, and have been linked to obesity This study explores the role, barriers, and motivators of takeaway food purchasing and consumption for families in England, and whether this differed by socioeconomic position (SEP).

**Methods:**

Inductive thematic analysis was conducted with a constructivist ontology lens. Participants were 20 higher and lower SEP parents/carers of school or nursery aged children across Bradford, Brent, and Folkestone and Hythe.

**Results:**

Both higher and lower SEP participants saw takeaways as a treat, a break from cooking, and a tradition. Lower SEP participants described these takeaway traditions as having begun when COVID-19 restrictions were introduced and expressed intentions to continue them. Higher SEP families used takeaways to try new cuisines and as a social event with the family. Price was a barrier for both higher and lower SEP participants, but only lower SEP families saw promotional offers as a motivator. Negative service experiences were a barrier for higher SEP participants. Both higher and lower SEP participants shared other family members buying takeaways as a motivator.

**Conclusions:**

This research suggests takeaways play a role in many families’ lives by producing structure, facilitating social events, and providing emotional support. These results contribute context and understanding of families’ food environments. As lower SEP families described being motivated by promotional offers, policy options to restrict the promotion of less healthy options should be explored.

**Supplementary Information:**

The online version contains supplementary material available at 10.1186/s12889-026-26585-0.

## Introduction

### Food environment and dietary health

The food environment plays a key role in shaping diet and long-term health outcomes across the life course [1]. Access to a balanced diet, rich in essential nutrients, is a cornerstone of non-communicable disease (NCD) prevention and healthy development [2]. However, modern food environments often prioritise energy-dense foods high in fat, salt, and sugar (HFSS) [1], creating structural barriers to meeting dietary guidelines [3]. With an ongoing cost-of-living crisis in the UK following COVID-19, and a growing number of families experiencing financial [4] and food insecurity [5], many with the lowest incomes are less able to prioritise long-term health when making food choices over immediate caloric necessity [6].

### Clinical consequences

Inequitable environments manifest in disparities in health outcomes, particularly in rates of overweight and obesity. Obesity, defined as excessive or abnormal bodily fat that may impair health [7], is an increasing global health issue [8], with half of the world’s population expected to live with overweight or obesity by 2035 [1]. Within the UK, almost two thirds (64%) of the adult population meet the criteria for overweight or obesity [9].

This presents a growing public health challenge, as overweight and obesity are risk factors for a range of non-communicable diseases, including cancer [10] and cardiovascular disease [11]. There is an increasing focus on obesity prevention as children living with overweight or obesity are at risk of maintaining this through to adulthood [12], putting them at an increased risk of future health problems. Further, childhood obesity can also create more short-term health effects, with estimates of up to 26.5% of children and adolescents living with obesity experiencing metabolic syndrome [13], and 20.5% of children and 31.6% of adolescents showing signs of pre-diabetes [14].

Of particular concern is the link between socioeconomic position (SEP) and obesity. Adults residing in the most deprived areas of England (measured by the English Index of Multiple Deprivation; IMD [15]) have higher rates of obesity (36%) compared to those living in the least deprived areas (22%) [16]. These inequalities often manifest in early childhood, with strong associations between neighbourhood deprivation and overweight and obesity when children are in the first year of primary school (aged 4–5 years) and when they are in the final year of primary school (aged 10–11 years) [17]. Further, the rise in childhood obesity during the COVID-19 pandemic was unprecedented, with prevalence up by 9.9% between 2019 and 2021 in the first year of primary school, and 21% in the final year of primary school. This rise also sharply widened the gap in inequalities, with those in the most deprived areas of England being twice as likely to live with obesity (12.9% and 29.2% in the first and final years of primary school) when compared to children in the least deprived areas (6% and 13% in the first and final years of primary school) [18].

### The food environment in COVID-19 and beyond

While the aetiology of obesity is complex and multifaceted, it is recognised that an increase in the consumption of energy-dense foods high in fat, salt, and sugar (HFSS), alongside a decrease in physical activity is associated with increased obesity risk. Obesogenic food environments play a key role in rapidly rising rates of overweight and obesity. Reduced access to fresh produce and a high density of fast-food restaurants and convenience shops leads to an environment where unhealthy eating becomes the default [19]. Concomitant to the pandemic, sales in the out-of-home food sector (food and drink prepared outside the home, including restaurants, cafes, and take-away outlets) dramatically rose, with Deliveroo, a UK-based online food delivery platform, reporting a 110% increase in orders [20] taking place between the implementation of strict lockdowns across 2020 through to the final lifting of all lockdown rules in July 2021. Takeaways are often energy dense and lacking in key nutrients, damaging to health, and linked to increases in obesity [21, 22]. With an estimate of close to 50,000 fast-food and takeaway businesses existing in England in 2024 [23], these foods are often also highly available. Takeaway exposure has been linked with increased consumption, higher body mass index (BMI), and greater odds of obesity [24], and may play a particular role in driving health inequalities. Indeed, there are five times more takeaway outlets in deprived areas of the UK when compared to affluent regions [25]. Further, children who live, work, and socialise within more deprived areas on average eat more fast food and have higher BMIs [26].

Several studies have explored the shifting role of food within family households during and post-pandemic, including reports of increased consumption in snacking and takeaways [27, 28]. Research has also shown that takeaway and fast-foods are often consumed by families for convenience and time saving, and as a tool for social bonding with friends and family [29]. However, no study to date has explored the role that takeaways played for families in different socioeconomic positions across England, and the contexts which influence takeaway consumption. With the food environment a politically salient topic for obesity policy, and the UK Government’s focus on prevention and reducing health inequalities in their 10 Year Health Plan [30], this study will provide context which policy advocates and makers can draw from to understand families’ experiences and differences across socioeconomic positions.

### Aims of this study

The aim of this study is to (1) understand the role of takeaways in the lives of families in England with nursery-aged or school-aged children, and differences between higher and lower SEP families; (2) understand the barriers and motivators for purchasing and consuming takeaways within these families, and whether this differs by SEP.

## Methodology

### Food in Lockdown study

#### Participants and recruitment

This study presents a secondary analysis of interview data collected for the Food in Lockdown study, which more widely explored the impact of COVID-19 of family’s relationships with food [31]. Sixty-two parents/carers of school- or nursery-aged children, up to the age of 16 years, were recruited across Bradford (northern English city), Folkestone and Hythe (government district in coastal south England), and the London Borough of Brent. These locations were chosen to provide a varying range of environments from urban city to coastal town surroundings.

Participants were quota sampled from social media channels, such as local Facebook groups and Twitter feeds, alongside snowball techniques through the request of participants to share information with others. Posters and a brief description of the study were provided on each post, including a link to the survey.

Participants were initially directed to fill in a short survey online or via telephone to capture key demographic data such as gender and ethnicity. SEP was determined using a composite index derived from a series of indicators, including occupation, postcode, and total annual household income, which has been previously validated for use in the UK [32]. Participants were asked if they had children of nursery or school age (up to age 16 years) but participants were not asked to provide details about their children. This demographic data was used for quota sampling [33] to ensure that participants were recruited from a range of ethnic and SEP backgrounds to reflect the diversity in their geographic location based on census data. If there was a gap in the recruitment bracket to meet these diverse reflections, participants that helped meet the quota were contacted to partake.

#### Semi-structured interviews

Interviews were conducted between April-June 2021. This temporal point was chosen as national lockdowns were lifting, restrictions eased, and schools and workplaces re-opened. This provided the opportunity to see how takeaways were viewed in light of difficult but re-normalising circumstances, whereby families would be returning to schools and workplaces. One interviewer (CGS) independently conducted interviews with participants, with all follow up interviews continuing with the same interviewer who facilitated the initial session. The interviewer was female, held an undergraduate university degree in psychology, and had experience in facilitating interviews. The interviewer was a research assistant at the time of the study with an interest in social and cultural experiences of health. Participants were not personally known to the interviewer and were informed of the aims of the study prior to taking part through an information sheet.

Participants could choose to take part in the interview over the phone or through a video conferencing platform such as Zoom. Interviews took around an hour to complete. An interview guide was piloted with the first three participants and iteratively edited based on the researcher’s interpretations of emerging topics of interest. Questions were designed to explore the participant’s food habits and environment, including discussions on takeaway use alongside school meals, cooking, and food shopping. See Additional File 1 for the full interview guide. Interviews were audio recorded using a digital recorder to be later transcribed. Fieldnotes were taken during and after interviews.

### Present study

#### Participants and recruitment

This study is a secondary analysis of qualitative interview data. The original participant pool consisted of (*N* = 60) parents and carers, who play a key role in shaping household food practices, decision-making, and day-to-day routines around food. For the current study, participants were selected by ranking the original interview pool by household income, selecting participants from the lowest income upwards and highest income downwards, to capture a broad spectrum of experiences and to identify contrasts, until researchers were unable to generate any new codes within either the high or low SEP groups.

#### Data analysis

Data were analysed in 2022. Thematic analysis [34] was chosen for analysing the transcripts, as this method is frequently used for describing, analysing, and reporting patterns in data. An inductive approach to data analysis was used [35] whereby no a priori themes were placed on the data prior to analysis; rather, themes were created through an interpretive process, reflecting the relationship between the researcher and the participants’ narratives. For this secondary analysis, the research team specifically focused on data relating to takeaway foods, while the original interviews explored broader dietary behaviours (e.g., school meals, cooking).

Analysis was completed by AS, who first familiarised themself with the data by reading the transcripts in multiple instances. Then, all transcripts from participants belonging to the lower SEP group were re-read before generating and collating codes based on data which related to the two main research questions. A codebook was generated and was used to guide the coding of higher SEP participants. Themes were then generated through identified patterns in the codes and refined through discussions with two other researchers (CGS, RC). Nvivo software (version 14) was used for analysis.

A constructivism ontology [36] was considered when analysing the data, in which the researchers recognised takeaways as social constructs. What is known as a ‘takeaway’ will vary across individuals and the contexts in which they appear in people’s lives will differ. Thus, the analysis aimed to focus on the roles participants ascribed to them through their use within family life, practices, and in decision-making contexts.

### Ethics

Ethical approval was provided by City, University of London Health Sciences Research Ethics Committee. All participants signed informed consent and the aims of the research were explained in detail prior to taking part in the study. All data were anonymised and identifying information within transcripts was deleted prior to analysis. The Consolidated Criteria for Reporting Qualitative Research (COREQ) [37] checklist was used to ensure transparency in reporting of the qualitative data.

## Results

### Demographics

A total of 20 parents/carers of school or nursery aged children were included in this analysis, 10 of whom had a household income ranging from £0 to £30,000, and 10 of whom had a household income of £37,000—£100,000 or above. The majority of participants were White British (55%), with a higher proportion in the higher SEP group (80%) than the lower SEP group (30%). All (100%) participants were female. See Table 1 for a breakdown of participant details.

**Table 1:** Lower and higher SEP participant demographics

Participant number	Household income	Ethnicity	Region	Spoke about takeaways
*Lower SEP participant demographics*				
1	£15,000—£22,000	Pakistani	Bradford	Yes
2	< £15,000	White Other	Brent	Minimally
3	£15,000—£22,000	White British	Bradford	Yes
4	£22,500—£30,000	Caribbean	Brent	Yes
5	£15,000—£22,000	Pakistani	Bradford	Yes
6	< £15,000	White British	Folkestone	Yes
7	< £15,000	White British	Brent	Minimally
8	< £15,000	Afghan	Brent	Yes
9	£15,000—£22,000	Pakistani	Bradford	Minimally
10	£22,500—£30,000	Pakistani	Bradford	Yes
*Higher SEP participant demographics*				
11	£37,000—£45,000	Asian and White	Bradford	Yes
12	> £100,000	White British	Brent	Minimally
13	£90,000—£100,000	White American	Folkestone	Yes
14	> £100,000	White British	Brent	Yes
15	£67,000—£75,000	White British	Folkestone	Yes
16	£67,000—£75,000	White British	Bradford	Yes
17	> £100,000	White British	Brent	Yes
18	> £100,000	White British	Bradford	Yes
19	£67,000—£75,000	White British	Bradford	Yes
20	£37,000—£45,000	White British	Bradford	Yes

## Research Question One: What was the role of takeaways for families within England during the COVID-19 lockdown?

Themes were largely shared across lower and higher SEP groups, however the context surrounding participants and how these roles were experienced differed across families. See Table 2 for a summary of themes.

**Table 2:** Summary of themes

Role of takeaways		
**Theme**	**Lower SEP participants**	**Higher SEP participants**
As a treat	Yes	Yes
Break from cooking	Yes	Yes
Tradition	Yes	Yes
Trying new cuisines	No	Yes
Social event	No	Yes

### As a treat

#### Lower SEP

Families saw takeaways as rewarding and of positive value, with many participants describing them as treats which were enjoyable for both parents and their children. For participant 1, takeaways were seen as something that was accessible and enjoyable for children when other options were limited or less appealing to children. For other families, takeaways were used as birthday treats for the parents and children, in lieu of other activities being unavailable during lockdown.

“*It’s something that the kids enjoy and at the minute it’s the only thing that we can really give them. Because they’re not interested in clothing or this that and the other. So I think that’s something that will continue, definitely.” (Participant 1)*

#### Higher SEP

Takeaways were seen as a treat for higher SEP families, although it was less spoken of. For some, these treats were used as a way to help alleviate emotions after difficult days. Treats from takeaways were often in the form of meals, such as buying dinner from a Chinese takeaway shop. For participant 15, however, a takeaway treat could be as simple as a coffee.

*"So, he’ll usually just go to the [restaurant] drive-thru and get us some coffees. So, it’s a bit of a treat on a Wednesday.” (Participant 15)*

### Break from cooking

#### Lower SEP

For some families, takeaways were seen as a crucial break for the parents while children stayed at home. However, they were not seen to be nearly as necessary when the children were back in school, although takeaways were still used infrequently to avoid cooking. For participant 1, takeaways were discussed as reducing the practical demands of substantial food preparation during Ramadan. The participant noted that their kitchen was too small for the large menu planned, and so takeaways provided a helpful alternative when hosting.. Thus, takeaways also appeared to function as a way of supporting hospitality and participation in culturally significant practices. These meanings were not elaborated in the interview, but are important for contextualising.

*“And even during Ramadan … Don’t get me wrong, my husband and son and daughter do help out in the kitchen, but you know when you’re preparing, it’s a small kitchen and when you’ve got so many things on the menu it becomes a bit of a nightmare sometimes.” (Participant 1)*

#### Higher SEP

Participants viewed takeaways as reducing the pressure of making decisions about what to cook every day and as time-saving. For participant 14, takeaways were used as a way of alleviating the boredom of cooking and as something that released them from their role as the main provider of meals for the family. Overall, takeaways were seen as creating a break from stressors.

*“…We started to have take-out a little bit more. I was just so fed up with cooking and food.” (Participant 14)*

### Routine

#### Lower SEP

For some families, takeaways had become part of a weekly tradition and was seen as an activity that could be done together as a family, although not many participants spoke of this theme. For participant 1, this was mostly on Friday because there would be no school the next day. For all lower SEP participants who saw takeaways as a routine, this had started during lockdown and was something they planned to continue.

*“So you have takeout with them. How often would that be?” (Interviewer)*

*“Once a week.” (Participant 5)*

*“And do you think if things go back to totally normal again, is that a tradition you’ll keep?” (Interviewer)*

*“Yes, once a week, definitely. I think it’s something you can maintain and it’s something that you do as a family” (Participant 1)*

#### Higher SEP

Many participants saw takeaways as being part of a routine. Most saw this as a weekend tradition that they ordered on a Friday or Saturday night. For some, this was often a routine where typically the same food would be ordered, such as fish and chips mid-week and pizza on a weekend. For participant 15, the weekend coincided with the time that they ran out of food in the house and so this was used as a prompt to order. For participant 13, who had emigrated from North America, they saw their routine takeaway consumption as quite low while their partner perceived it to be high, due to cultural differences.

### Trying new cuisines

Lower SEP participants did not state that takeaways played a role in trying new cuisines.

#### Higher SEP

Some families used takeaways as a way to teach their children about different tastes, cultures, and textures. For some, this started just before lockdown and continued throughout. For participant 11, trying new cuisines was a scheduled event once a month where they would discuss what they would choose and why. For another, delivery options allowed them to expand their children’s tastes to foods such as sushi and Chinese cuisines.

*“We put it in the diary, we have a discussion in what we choose and why we choose it. […] We’ve tried to do it more because it was one more thing that I wanted to teach [13 year old son] to taste different cuisines” (Participant 11)*

### Social event

Lower SEP participants did not state that takeaways played a role in social events.

#### Higher SEP

Some participants saw takeaways as a social event with their partner or children. Participant 19 described ordered takeaways alone but making orders more frequently when their husband was home. For another, ordering a takeaway only happened if their partner was in, as it was seen as a shared experience. In another example, participant 17 ordered takeaways to share with their daughter when their partner was not in, seeing it as *“just a girly thing”* and to *“get something maybe dad wouldn’t have”.*

## Research Question Two: What were the barriers and motivators for families regarding takeaway purchasing and consumption?

Higher and lower SEP participants shared price as a barrier, and family members as a motivator, for ordering takeaways. However, only higher SEP participants spoke of previous poor experiences as a barrier to re-ordering, and only lower SEP participants described promotional offers as a motivator. See Table 3 below for a summary.

**Table 3:** Summary of themes relating to barriers and motivators

Barriers and Motivators		
**Theme**	**Lower SEP participants**	**Higher SEP participants**
High price	Yes	Yes
Poor experiences	No	Yes
Promotional offers	Yes	No
Other family members	Yes	Yes

### High price

#### Lower SEP

Price was the only barrier noted by lower SEP participants. While takeaways were regarded as rewarding, perceptions of their value differed depending on the price associated with them. Participant 3 stated that while they had previously enjoyed ordering takeaways regularly, their financial position had changed which meant they had recently reduced the number of takeaways they ordered as they could no longer afford them. For this family, ordering takeaways was often seen as “wasted money” which could be put towards more cost-effective purchases, such as grocery shopping which could produce multiple meals for the same price.

*“So, it was just a waste of money really. I could have done a little shop with that and got a fair few meals out of it. So, I want to stick to not having so many takeaways.” (Participant 3)*

For *participant 1*, ordering both a main meal takeaway and a dessert was seen as something that “you can’t maintain” in the long-term. The alternative for this participant was to have homemade desserts to save on the cost while still being able to order a substantial part of their meal.

Some takeaway meals were observed to be served in smaller portions and become increasingly more expensive during lockdown, leading to the perception that they were not good enough value for money to continue purchasing. This led participants to only choose from cheaper establishments or where there were offers available.

*“But [chicken restaurant], they love their meals, but their portions are getting smaller and the prices are going higher. Typically for five of us, even with the 25% off you’re looking at £40 to £50 a meal. Where with [restaurant] you can pay £25 and get it.” (Participant 1)*

#### Higher SEP

Participant 17 spoke of price as a barrier to ordering takeaways when cooking was seen as a cheaper, alternative option. For this participant, takeaways were previously stated to be used as a break from cooking when time was an issue, and so price may only be a barrier when it is weighed up against other competing factors.

*“I sort of reached the point where I’m like, maybe I’d rather just cook than spend all that money.” (participant 17)*

### Poor experiences

#### Lower SEP

No lower SEP participants spoke about poor experiences as a barrier to ordering takeaways.

#### Higher SEP

Previous, negative experiences of takeaways impacted participant’s willingness to re-order, and for participant 17 this tied in with issues of price in relation to quality when compared to home-cooked food.

*“…ended up with [restaurant] which was just pretty grim really. I object for paying that much money for something I could cook better at home but [the children] were very happy.” (Participant 17)*

For another family, issues with the delivery of the food created a negative perception whereby money was exchanged but the order never arrived. This led to a lack of motivation to want to order again.

*“During the lockdown we ordered once from [takeaway] and we kept phoning them asking where the food was, and they kept lying to us […] we didn’t see the food and we haven’t seen our money.” (Participant 11)*

### Promotional offers

#### Lower SEP

Some families saw promotional offers as a motivation to order, as deals were often perceived as good value for money. For some families, parents would give their children options of establishments to order from, with the choices determined by whether offers were available or not.

*“I’ve given them options of, you can pick from here, there, or there based on offers.” (Participant 1)*

Promotions were seen as something to take advantage of when they were available, with repeat orders given to the same establishment because of the perceived value for money.

*“UberEATS app online had their promotional offers, there was one that became a favourite of ours. [Restaurant] did a buy one get one free. So where you were paying £13 for a Parmesan or £13 for half a Piri Piri chicken meal, it was half price. So that was something I definitely took advantage of.” (Participant 1)*

For others, when they were choosing between different establishments, deals played a critical role and were key to the decision to choose one takeaway over another.

*“… And they were doing a deal, so we had that.” (Participant 6)*

#### Higher SEP

Higher SEP participants did not state promotional offers as being a motivator for purchasing takeaways.

### Other family members

#### Higher and lower SEP

For some participants across both SEP groups, other family members such as their children’s grandparents, aunts or uncles often invite the children to have takeaways with them.

“My family, they invite my children as well because they know that they're fond of takeout.” (Participant 5, lower SEP)

*“…Then the grandma came and bought us [fast food restaurant].” (Participant 11, higher SEP)*

For one lower SEP family, the ex-partner of the participant—who is the father of their child—provided takeaways when picking his child up from events against the wish of the participant.

*“So, if he [dad] picks them up from tuition, he’ll just take them to [restaurant], and then they’ll probably eat in the car … It's hard trying to be the parent who’s influencing [to eat healthier]… stopping them having [restaurant].” (Participant 8, lower SEP)*

## Discussion

This study explored the role of, and barriers and motivators for purchasing and consumption of, takeaways in higher and lower SEP families during the COVID-19 pandemic. Themes were largely shared across higher and lower SEP participants, whereby takeaways were seen as a treat, a break from cooking, and a tradition. However, only higher SEP families spoke of using takeaways to expose their children to new cuisines and as a social event. Barriers of price were spoken of by both higher and lower SEP families, but only lower SEP families described promotional offers as a key motivator. These results suggest that takeaways play a prominent role in everyday family life and also point to differences across socioeconomic positions regarding why takeaways may be purchased and consumed. We note a small possible overlap between concepts such as “a treat” and “social event,” as occasions such as birthdays can be considered in both, and participants may frame the same event in more than one way.

COVID-19 brought considerable changes to the food environment and families’ living circumstances, with many children and parents home from school and remote working. The experiences discussed in interviews reflect the changes that many made to adapt to changing circumstances during COVID-19: takeaways provided a structure, often weekly, for families across socioeconomic positions where previous structures had been removed. Research suggests that, during COVID-19, the out-of-home sector pivoted from a mostly convenience-based model to a form of household entertainment and leisure during lockdowns, with the ‘special’ dining experience of a restaurant entering into the home [38]. Indeed, in a survey exploring experiences during the pandemic 62% of adults saw food as ‘family time’, and 51% said it was a ‘luxury to treat yourself’ [39].

The role of takeaways in providing routine was similar for both sets of participants, where takeaways were often seen as weekly traditions with specific cues, such as Friday evenings when grocery supplies had been depleted in the house. For many, this routine began in lockdown and for all lower SEP participants who spoke of this, it was something that they intended to continue as restrictions eased. With takeaways often being calorie-dense and low in key nutrients [40, 41], and higher consumption being linked to higher BMI and body fat percentage [19], a permanent shift in behaviour towards regular takeaway consumption may have negative implications for rates of overweight and obesity. Indeed, revenues have continued to grow for most food delivery app companies post-COVID, and the entire industry is expected to reach a market size of $213 billion by 2030 [42].

Only higher SEP participants spoke about takeaways playing a role in giving their children new cuisines to try, such as by introducing them to sushi. Families saw takeaways as an opportunity to introduce their children to different tastes, cultures, and textures; for participant 11 this was a scheduled and structured event once a month. This aligns with survey data suggesting that over a third of adults had eaten at or ordered from a takeaway that they had never visited pre-pandemic [32]. It is possible that these discussions served as a form of justification for ordering takeaways, with participants framing their consumption as educational to maintain social desirability. It is also possible that healthier takeaway options, such as sushi spoken of by higher SEP participants, were prohibitively expensive and/or less accessible for those in the lower SEP group, which could have implications for overweight and obesity rates across SEP. Further, food delivery apps provide delivery premiums and service fees which may restrict lower SEP families to more local, walk-in businesses compared to the wide range and variety available on some apps. This may widen inequalities, given that a recent modelling study found out-of-home food outlet menu healthiness to be socioeconomically patterned, with more outlets with unhealthy menu options found in more deprived areas in the UK, relative to less deprived neighbourhoods [43].

Rates of parental burnout and emotional distress were increased during the pandemic, particularly for families with lower incomes [44]. In our sample, only higher SEP participants described using takeaways on difficult days. It would be of interest to further study the role takeaways play in times of instability, particularly with global instability rising amid armed conflicts, extreme weather events, and geoeconomic confrontation, [45], and whether patterns are evident across varying socioeconomic positions. Indeed, many families discussed using takeaways as a treat, some after difficult or emotional days, and thus takeaways are likely to continue to have a role as a self- and family-soothing activity, with 64% of UK adults describing food as a source of comfort when surveyed during the pandemic [33].

Higher and lower SEP participants shared the theme of family members buying takeaways for them and their children. However, only higher SEP participants spoke of previous poor experiences as a barrier to re-ordering, whereby unpleasant meals or delivery services in the past meant they did not engage as much with takeaways now. This suggests that for higher SEP participants, quality of experience may be a primary motivator for ordering takeaway foods, whereas for lower SEP groups, the functional role of the meal, satiety and value, may take precedence.

High price was a barrier to ordering takeaways for both higher and lower SEP participants, but it was spoken of more frequently by the lower SEP participant group and was seen as wasted money and unsustainable purchasing. For higher SEP participants, price being a barrier was often relative to how much cooking was seen as an effort; in contexts where it seemed less effortful to cook, the price was often unjustified. Takeaways were stated by higher SEP participants to be used as a break from cooking when time was an issue, and so price may only be a barrier for some when it is weighed up against other competing factors.

These findings highlight a possible gap in agency for lower SEP participants within the food environment: when families do engage with takeaways, they may be more susceptible to volume-based promotions and value deals for energy dense foods, to help mitigate against the price barrier.

The finding that promotional offers were described as a motivator for purchasing takeaways and choosing between different options among lower, but not higher, SEP families is important when considering policy options to reduce health inequalities. Lower SEP families often chose where to order based on the promotions they offered and saw these meals as better value for money. As the UK Government aims to introduce new barriers to promotional offers within supermarkets for HFSS foods in order to curb obesity [46], the findings from this study may have implications for wider policies that may seek to extend these restrictions to restaurant and takeaway businesses.

As inflation grew across 2023 and grocery prices rose exponentially, the UK entered a ‘cost-of-living crisis’ [47] which has since continued. This crisis has seen many families experiencing financial hardship and is likely to impact on food purchasing [14]. Lower SEP families are in less of a position to prioritise long-term health and instead opt for cheaper, more calorie-dense foods during economic hardship [48] as healthier and affordable food becomes less accessible and more families move into a food-insecure environment [49].

Promotional offers and cheaper takeaways may have offered higher purchasing incentives for lower SEP participants during COVID-19 and this pattern is likely to continue during the cost-of-living crisis. Partnered with the growth in higher availability of takeaways in lower income areas, this research may highlight reasons for future widening inequalities in overweight and obesity across different socioeconomic positions. With the UK Government’s 10-year Health Plan [27] placing emphasis on prevention and reducing inequalities, there is a growing need for targeted policies that address structural barriers to healthy eating for vulnerable households.

### Limitations

This study is not without its limitations and future research should be conducted to investigate the implications of this research further. Secondary analysis limited the ability to probe further into details relevant to the specific research aims of this study, and thus future research based on these emerging findings would help to strengthen these results and enhance our understanding of the uncovered themes. For example, as a secondary analysis of data, it was not possible to investigate if the same motivation for promotional offers is shown when applied to healthier foods, such as fresh supermarket produce, and whether this would be reflected in both higher and lower SEP families. Further, we were not able to explore in detail the nutrient profiles of the takeaways bought by families in lower and higher SEP groups. This may be of particular interest to investigate when considering future policy options to reduce obesity within England.

While this study aimed to recruit a diverse population in relation to ethnicity and SEP, the use of quota sampling may provide a limitation to the results and introduce sampling bias as the researcher determines the sample. In the future, stratified sampling may be more appropriate to produce a representative but randomly selected sample of participants [50]. Further, the perspectives offered in this study are narrowed to parents and carers of nursery or school-aged children and thus may not reflect the experiences of families with older children or different household structures. As all participants in this study were female, the results should be interpreted within this specific context. Future research including male and gender-diverse participants would be beneficial to determine if the underlying motivations for using takeaways differ across male and gender-diverse parents.

The data in this study may have been subject to social desirability bias from the participants, whereby some may have under-reported their takeaway frequency due to perceived stigma, or been subject to selective recall from incorrect self-reporting of takeaway consumption.

It is also essential to acknowledge the researcher’s positionality within the data analysis. The interpretation of the themes are filtered through the researchers' own socioeconomic and cultural lenses, which may influence the extent to which the findings fully represent the lived experiences and cultural nuances of the participants.

### Conclusion

In conclusion, this research suggests that the role that takeaways play for families in England during COVID-19 was often shared across socioeconomic positions, often being seen as a treat or as part of a weekly routine. Some themes, such as social events and trying new cuisines, were only spoken of by higher SEP participants. Further, promotional offers appeared to motivate lower, but not necessarily higher SEP families to purchase takeaways or choose one takeaway over another. These results suggest that takeaways play a role in many families’ lives by producing structure, facilitating social events, and providing emotional support during difficult times. This research contributes context and understanding of the experiences of families during COVID-19 and has ongoing implications for the development of inclusive policies, in order to help narrow health and obesity inequalities across socioeconomic positions. More research should be conducted to investigate these themes further in relation to potential policy interventions, such as creating promotional offers for healthier produce, and how responses may differ across SEP.

## Supplementary Information

Additional file 1: Title of the data: Full interview guide. Description of the data: A full interview guide for the Food in Lockdown study.

## Data Availability

The datasets generated and/or analysed during the current study are not publicly available due to protecting the anonymity of interview participants, but an anonymised version may be made available from the corresponding author on reasonable request.

## Abbreviations

BMI: Body mass index
HFSS: High fat, salt, and sugar
SEP: Socioeconomic position
