# Supplementary material for: Understanding the role, barriers, and motivators of takeaway food purchasing and consumption in English families across socioeconomic positions: a secondary qualitative analysis
Source: BMC Public Health. 2026 Feb 12;26:912. doi: 10.1186/s12889-026-26585-0 (PMC12997662; doi:10.1186/s12889-026-26585-0)
Supplement: Supplementary file 1 — Additional file 1: Title of the data: Full interview guide. Description of the data: A full interview guide for the Food in Lockdown study. [file 12889_2026_26585_MOESM1_ESM.docx]

**Additional File 1:** Full interview guide for the Food in Lockdown Study.

**Section 1: life since lockdown, health and wellbeing**

Acknowledgement of the challenges of the past six months and all of the things that have changed.

**The family**

- How has everyone in your family been since we last chatted?
- Probe for any significant changes
- Did anyone have COVID?
- Did you have to self-isolate at any point?

If yes, probe for the experience of this and any related financial/ food implications

**General experience of lockdown**

I’m interested in what day to day life for your family looked like during the January to March lockdown?

Probes:

- In what ways did your day-to-day life change?
- Any work changes?
- New challenges?
- Have there been any positives?
- How have you managed financially?
- What does day to day life look like now?
- How does it feel now we can start to see people again/ go to different activities?
- Are there things you’re concerned about as we enter into this next phase of normal?

If financial challenges are disclosed

- How has this impacted your day to day life?
- How has this impacted on you being able to afford and buy the things you and your family need?
- Would you say this has impacted the kinds of foods you eat?
- Have you been able to receive any support from the government?

**COVID winters grants; self-employment grants; school support.**

- How do you feel about this support?
- How has [this challenge] impacted you?
- Are you eligible for healthy start?

If underlying health condition in family disclosed

- Have you been able to receive any support from the government during lockdown?
- How did you feel about this support?

**The area**

- Has much changed about [neighbourhood] in the past six months?
- Are you still getting around by [transport mode]

**Perceptions of health and wellbeing**

In the last interview we spoke a lot about health and wellbeing and what those terms meant? Do you think since the lockdown you’ve been thinking any differently about what those things mean?

- Is there anything in that’s changed in terms of how you keep healthy and well in the past 6 months?
- Can you tell me about those changes?

Probe into the wellbeing and health chart

**Section 2: Engagement with the food environment**

**Shopping routines**

When we spoke six months ago, you mentioned shopping looked like x. Did your routines change much with the January lockdown?

Probes:

- Who did the shopping?
- In what places? Why?
- How often?
- Did your children go with you?
- How did you travel there?
- How did you navigate childcare and going shopping?
- Did you still shop to support x? Did you shop for anyone else.
- What does shopping look like now things have eased up a bit
- How have you felt about food shopping during the last lockdown?

Probes:

- Did you enjoy it more or less?
- Were there any changes to your routines that you would want to hold on to?
- Were there things you wish could be different?
- How easy or difficult dDid you find it to find time to get to the shops?
- If eligible for healthy start (child >4 yrs and earning >£16,190/ child tax credit / on UC / income support)
- How do the healthy start vouchers fit in?

Probe:

- - Where would you use healthy start vouchers
  - What do you buy with them
  - What do you think of the scheme?
  - Do you find that they help you to buy the things you ideally would want to?

**In-store practices**

When we spoke you said you thought about x when deciding what to buy. Is that still the case?

- If no, probe for reasons why
- What about how you go around a supermarket? Other store?
- Did you do anything differently in
- January?
- Has the layout of shops changed at all in the last 6 months?

Probe:

- If so, how?
- How do you find these changes to the layout?
- Do you buy the same things as you were buying six months ago or has anything changed?
- Can you tell me about those changes?
- You said before you did/ didn’t get things you weren’t planning to. Has this changed at all? Can you think of any reasons for that?
- Have you noticed any changes in the price of food since we last spoke?
- If so, has this influenced what you buy?
- Have you noticed any changes in food advertising?

Probe:

- In-store
- Outside
- Digital (on TV/ on social media & online)

If you could create a new place to get food in your local area, what would it look like?

If ordering online is mentioned:

Is this new since January months?

- What do you think of ordering online?
- Will you continue to do so post COVID? Why?
- Are there certain things you would or wouldn’t order online?

- If haven’t been able to order online since COVID but used to:

- Would you choose to start ordering online again?

If challenges in terms of finding to time to shop due to front line role, caring responsibilities

- Can you tell me a bit more about how that experience has been for you these past few months.
- What would have made things easier?

**Eating out and takeaway**

You mentioned last time that x about takeaway and eating out. Did this change in January? How has this changed? For what reasons would you have gotten takeaway?

- If they ate out regularly:
- What do you want it to look like in future?

**Section 3: Food practices**

Ideal mealtimes

When we last spoke you told me x about your idea mealtime and how x was important to you. How closely have things looked like that since January

**Meal preparation**

When we last spoke you told me x prepared most of the food. What do things look like now?

- If a change: How do you feel about that change?
- Has anything changed about the food/ meals you prepare in the last 6 months?

Probes:

- More or less from scratch cooking – why?
- More experimenting with new recipes – why?
- More baking – why?
- More cooking or eating as a family – why?
- More ready meal preparation – why?
- Planning your meals in advance?
- So it sounds like you are doing more/less since January. How has that been for you?/ What has that been like for you?
- Do you store food any differently to before? Why?

Probes:

- Freezing more
- Buying more long life foods
- Throwing food away?

Are there things you have changed that you’d like to keep?

**Meal times**

What did daily meals during the Jan-March lockdown?

- How do you feel that?
- Do you tend to eat together?
- If changed, are these changes you’d like to keep as things get back to ‘normal’
- Do you and your family members still eat food in between formal meal times/ outside of these formal meal times?
- What kind of foods do you eat in these times?
- What’s usually the reason for eating outside of these meal times?

**Types of food consumed**

[if not mentioned previously] Do you think that you (and others at home) have eaten any differently during the Jan-March lockdown?

- Can you describe those differences to me?
- Why do you think you and your family are doing these things differently?
- How do you feel about these changes?
- What do you think about the idea of these changes continuing into the future?

Additional probes:

- More or less fruit and veg (types)- why?
- More or less snack food (types)- why?
- More or less HFSS food specifically (types)- why?
- More or less out of home food (types)- why?
- More or less sugary drinks? – why?
- More or less energy drinks? – why?
- More or less healthy?

Is there anything you’d like to change about what you and your children eat?

How much influence do you feel you have over what your kids eat? Has this changed since lockdown?

Is there anything that you want to continue as things get back to ‘normal’ in terms of how you cook and eat? (eating together, cooking from scratch, shopping in different outlets, n. of takeaways, baking)

Thinking about the role food plays in your day to day life, would you say that’s changed much in the past 6 months?

**Section four: school food**

What has eating at school/ nursery been like since March?

Probes:

- Do they have school meals?
- What do they think of them?
- What do you think of them?
- Do they get water at school? How accessible is this? How popular is this?
- Did they ever go and buy extra food outside school?
- Has this changed in the past six months?
- Did the school offer any support during the Jan-march lockdown?
